# Post‐Stroke Depression and Determinants in Ethiopia; A Systematic Review and Meta‐Analysis

**DOI:** 10.1002/mpr.70109

**Published:** 2026-10-12

**Authors:** Getanew Kegne Nigatie, Berhanu Wale Yirdaw, Getaw Wubie Assefa, Worku Chekol Tassew

**Affiliations:** ^1^ Department of Reproductive Health Teda Health Science College Gondar Ethiopia; ^2^ Department of Pediatrics and Child Health Nursing Teda Health Science College Gondar Ethiopia; ^3^ Department of CTID and HIV Medicine Teda Health Science College Gondar Ethiopia; ^4^ Department of Medical Nursing Teda Health Science College Gondar Ethiopia

**Keywords:** determinants, Ethiopia, meta‐analysis, post stroke depression

## Abstract

**Introduction:**

Post stroke depression is a critical barrier to recovery and rehabilitation, contributing to a higher risk of severe disability, cognitive and functional decline, greater dependence on daily activities, and poor adherence to treatment. Additionally, post‐stroke depression is a leading cause of death globally. The prevalence of PSD varies significantly depending on the type of study and screening methods used. However, it remains poorly understood and inadequately explored in many parts of the world, especially in developing countries. To identify the key determinants of PSD, conducting a systematic review and meta‐analysis is crucial and relevant to current clinical practices.

**Methods:**

In conducting this review, the International Preferred Reporting Items for Systematic Reviews and Meta‐Analyses checklist was used. This review focused on studies examining the prevalence of post‐stroke depression and its determinants in Ethiopia. Two authors conducted electronic searches in PubMed, Google Scholar, Science Direct, and African Journals Online Library, with the search period extending until April 8, 2024. Data were extracted using Microsoft Excel and exported to STATA version 11 software for analysis. Heterogeneity of studies was assessed by Cochran's Q test and I^2^ statistics.

**Results:**

The analysis included the final eight studies. The pooled prevalence of post stroke depression was 40.9% (95% CI: 26.52–55.28, *p* < 0.001). The analysis revealed significant heterogeneity among the included studies (I^2^ = 91.1%, *p* < 0.001). Being female (OR 3.51, 95% CI: 1.87–6.58), being illiterate (OR 2.02, 95% CI: 1.29–3.17), and being over 40 years old (OR = 1.59, 95% CI: 1.09, 2.32) were determinants of post stroke depression.

**Conclusions:**

According to the current review, post stroke depression is highly prevalent in Ethiopia. Being female, being illiterate and being over 40 years old were factors associated with post stroke depression. Therefore, attention should be given to strengthening social support; health professionals should focus on reducing disability/physical dependency and depression, as these are vital factors for improving quality of life.

## 1 Introduction

Stroke is also referred to as cerebrovascular accident caused by the interruption of blood circulation to the brain (Sacco et al. 2013). Globally, cerebrovascular accident is the second and third most common cause of death and permanent impairment, respectively (Feigin et al. 2022). Due to its complex management and devastating complications, stroke significantly affects the functional performance and daily life of patients (Choi et al. 2023). Around 15 million people worldwide suffer from cerebrovascular accidents each year, with one‐third of them passes away and five million develop long‐term impairment (De Ryck, Brouns, et al. 2014). According to a worldwide assessment, approximately 17 million cerebrovascular accidents occurred by 2010, nearly two‐thirds of these incidents take place in developing countries (Mukherjee and Patil 2011). A global increase in the magnitude of cerebrovascular accidents is devastating, with a high socioeconomic burden (De Ryck, Brouns, et al. 2014). According to the 2019 report, cerebrovascular accidents are the fifth most common cause of death in Ethiopia. The burden of cerebrovascular accidents in Ethiopia is an emergent neurologic problem (Yan et al. 2016).

Vascular conditions like stroke have been shown to play a significant role in the development of mental health comorbidities. After a stroke, mental health disorders can emerge due to either direct brain injury or maladaptive reactions to the stroke's aftermath (David et al. 2012). Currently, mental health problems are significantly increasing among stroke patients (Jose 2013). Common psychiatric problems that arise after cerebrovascular accidents include psychotic disorders, stress, and mood disturbances (Rao 2011). Post‐stroke depression (PSD) is a common and impactful mental health problem following cerebrovascular accidents, significantly influencing a patient's overall prognosis in a negative way (Hackett and Pickles 2014). Patients who are victims of a mild stroke are vulnerable to several invisible and neglected neurological complications. In parallel, it is known that fatigue and neuropsychiatric symptoms are common complications after a stroke in general (Vitturi et al. 2021).

Post stroke depression is a critical barrier to recovery and rehabilitation, contributing to a higher risk of severe disability, cognitive and functional decline, greater dependence on daily activities, and poor adherence to treatment. Additionally, post‐stroke depression is a leading cause of death globally (Das and Rajanikant 2018; De Ryck et al. 2013; Ellis et al. 2010). Post stroke depression can cause severe physical and functional impairment. Patients with significant functional impairments, longer hospital stays, poorer rehabilitation outcomes, lower quality of life, and higher mortality rates, particularly within the first 12 months, often experience slower or incomplete recovery (Wei et al. 2015). The prevalence of PSD varies significantly depending on the type of study and screening methods used. However, it remains poorly understood and inadequately explored in many parts of the world, especially in developing countries (De Ryck, Fransen, et al. 2014; Ojagbemi et al. 2017).

While earlier studies have yielded inconsistent results, PSD has been associated with various factors such as age, gender, ethnicity, cultural background, and education level. At present, the most consistent predictors of PSD are physical disability, stroke severity, previous history of depression, and functional limitations (Kaadan and Larson 2017; Towfighi et al. 2017). Studies on PSD is not adequate in African countries compared to Western nations. Given the differences in socioeconomic status, culture, and access to quality stroke care between developing countries and the West, the factors influencing PSD may vary in the Ethiopian context. However, there is a lack of detailed information and limited focus on neuropsychiatric conditions like PSD, which are often overshadowed by physical impairments. The goal of this review was to assess the prevalence and determinants of depression after stroke in Ethiopia. To identify the key determinants of PSD, conducting a systematic review and meta‐analysis is crucial and relevant to current clinical practices. Taking preventive measures, promoting early assessment, ensuring proper management, and improving the quality of life for patients would be the most effective approach.

Although several primary studies have investigated post‐stroke depression among stroke survivors in Ethiopia, evidence from individual studies varies considerably in terms of study setting, sample size, and reported prevalence. Therefore, synthesizing the available evidence through a systematic review and meta‐analysis is important to provide a more comprehensive estimate of the burden of post‐stroke depression in Ethiopia. To our knowledge, there is limited evidence from nationally focused meta‐analytic studies that comprehensively summarize the prevalence of post‐stroke depression among Ethiopian stroke survivors. The present systematic review and meta‐analysis was therefore conducted to estimate the pooled prevalence of post‐stroke depression and to explore potential sources of variation among the available studies.

## 2 Material and Methods

In conducting this review, the International Preferred Reporting Items for Systematic Reviews and Meta‐Analyses (PRISMA) checklist was used. This systematic review and meta‐analysis was conducted following the protocol that was registered with International Prospective Register of Systematic Reviews (PROSPERO) (Reference No: CRD42025632137) (Shamseer et al. 2015).

## 3 Search Strategy for Articles

This review focused on studies examining the prevalence of post‐stroke depression and its determinants in Ethiopia. Two authors conducted electronic searches in PubMed, Google Scholar, ScienceDirect, and African Journals Online Library, with the search period extending until April 8, 2024. A search was performed with the following keywords: “depression,” “post‐stroke depression,” “mood disorder,” “common mental problems,” “depressive disorder,” “stroke,” “brain attack,” “cerebrovascular accident,” “determinants,” “risk factors,” “predictors,” and “Ethiopia.” The search was conducted by two authors (WCT and GKN). Additionally, we explored further studies by reviewing the references of the retrieved articles through various databases (Table 1).

**TABLE 1:** Example of PubMed search history for prevalence of post‐stroke depression and determinants.

Search	PubMed search results	Hits
1	Depression OR post stroke depression OR mood disorder OR common mental problems	1869
2	Stroke OR brain attack OR cerebrovascular accident	3404
3	Determinants OR associated factors OR predictors	3632
4	#1, #2 and #3	1262
5	Limits; articles done in humans, published in English language, conducted among adults and free full text	4

## 4 Eligibility Criteria

This systematic review and meta‐analyses included articles that met the following specific inclusion criteria (Sacco et al. 2013): observational studies such as cross‐sectional, cohort and case control studies that report the prevalence and associated factors of post stroke depression (Feigin et al. 2022); studies conducted in Ethiopia (Choi et al. 2023); only articles that had been published (De Ryck, Brouns, et al. 2014); articles published until April 8, 2024 (Mukherjee and Patil 2011); articles published in English; and (Yan et al. 2016) articles conducted either in community or health facility settings. Articles lacking full text, unpublished studies, low‐quality studies, case reports, qualitative studies, conference papers, as well as systematic reviews and meta‐analysis were excluded.

## 5 Dependent Variable

The dependent variable of the study was the pooled prevalence of post‐stroke depression. Diagnosis of PSD was assessed by questionnaires adopted from Patient Health Questionnaire version nine (PHQ‐9). Clinically significant depression was considered when the PHQ‐9 is 10 and above. All people who have experienced a stroke should be considered at risk for post‐stroke depression, which can occur at any stage of recovery. All people who have experienced a stroke should be screened for post‐stroke depression if deemed medically appropriate.

## 6 Article Selection and Data Extraction

Articles identified through the search process were imported into EndNote software version X7 (Thomson Reuters, New York, NY), and duplicates were eliminated. The titles of the remaining studies were screened by two authors (WCT and GWA), and articles that did not fulfill the inclusion criteria were excluded. Prior to data extraction, the full texts of the selected article were assessed by two authors (WCT and GWA) against the eligibility criteria. Studies that met the inclusion criteria were used to extract data. Two authors (BWY and WCT) used Microsoft Excel to extract data from the included studies using a standard data extraction template. The following study features were included in the data extraction form: study design, sample size, participants or response rate, sampling technique, tools utilized, corresponding author name, publication year, study setting or region, prevalence of depression after stroke, and determinants.The disagreements between the authors were resolved by group discussion.

## 7 Quality Assessment

The Johanna Briggs Institute (JBI) quality evaluation checklist for analytical cross‐sectional study was used to evaluate the quality of articles that were included in the final analysis (Institute 2017). The following nine items are included in the JBI quality assessment checklist (Sacco et al. 2013): was the intended population adequately served by the sample frame (Feigin et al. 2022)? Were study participants properly sampled (Choi et al. 2023)? Was the sample size sufficient (De Ryck, Brouns, et al. 2014)? Were the study participants and the setting thoroughly described (Mukherjee and Patil 2011)? Was the identified sample adequately covered by the data analysis that was done (Yan et al. 2016)? Were appropriate techniques employed to diagnose the problem (David et al. 2012)? Were all subjects measured using a consistent, accurate method (Jose 2013)? Was a suitable statistical analysis conducted (Rao 2011)? Was the response rate adequate?

The overall methodological quality of the study is determined by the total score (ranging from 0 to 9), categorized as low (0–4), medium (Mukherjee and Patil 2011; Yan et al. 2016), or high (David et al. 2012; Jose 2013; Rao 2011) quality.

## 8 Statistical Analysis

### 8.1 Heterogeneity Test

Data were extracted using Microsoft Excel and exported to STATA version 11 (STATA Corp. LLC) software for analysis. Heterogeneity of studies was assessed by Cochran's Q test and I^2^ statistics. A *p*‐value less than 0.05 represent the presence of statistically significant heterogeneity among the included studies Heterogeneity was classified as no (I^2^ = 0.0%), low (I^2^ < 25%), moderate (25% ≤ I^2^ ≤ 75%), or high (I^2^ > 75%) (Huedo‐Medina et al. 2006; Cumpston et al. 2019). Due to the presence of Heterogeneity, among the included studies, a random‐effects model was employed to estimate the pooled prevalence of post stroke depression. Specifically among the methods of random effect, Der‐Simonian–Laird random‐effects models was used to generate the pooled prevalence of post stroke depression (George and Aban 2016). To identify the source of heterogeneity between the included studies, subgroup analysis was done using region and sampling technique. Sensitivity analysis was also performed to check the effect of each study on the overall prevalence.

### 8.2 Publication Bias

Publication bias was detected by the visual inspection of the funnel plot and statistical Egger's test. A *p*‐value less than 0.05 from Egger's test indicated notable publication bias, implying that studies with larger effects (either positive or negative) are more likely to be published (Rendina‐Gobioff 2006). Subgroup analyses were conducted to explore potential sources of heterogeneity among the included studies. Studies were stratified according to relevant study characteristics. The pooled prevalence of post‐stroke depression was estimated separately for each subgroup, and the results were compared to assess whether differences between subgroups could explain some of the observed heterogeneity. Subgroup analyses were considered exploratory because of the limited number of available studies.

## 9 Results

### 9.1 Study Selection

A total of 15,899 articles were found by the database search. A total of 15,500 of these articles were retrieved from African Journal Online, 4 from PubMed, 391 from ScienceDirect, and 4 from Google Scholar. After reviewing the titles, we removed 10,820 articles due to duplication. We screened the remaining 5079 articles and excluded 5051 because they did not focus on stroke patients. We assessed the eligibility and quality of the remaining 28 studies. However, due to the outcome variable not being reported and the studies being conducted outside of Ethiopia, additional articles were excluded. The analysis included the final eight studies. All the included studies used the Patient Health Questionnaire version nine (PHQ‐9) to assess post‐stroke depression (Figure 1).

**FIGURE 1:**
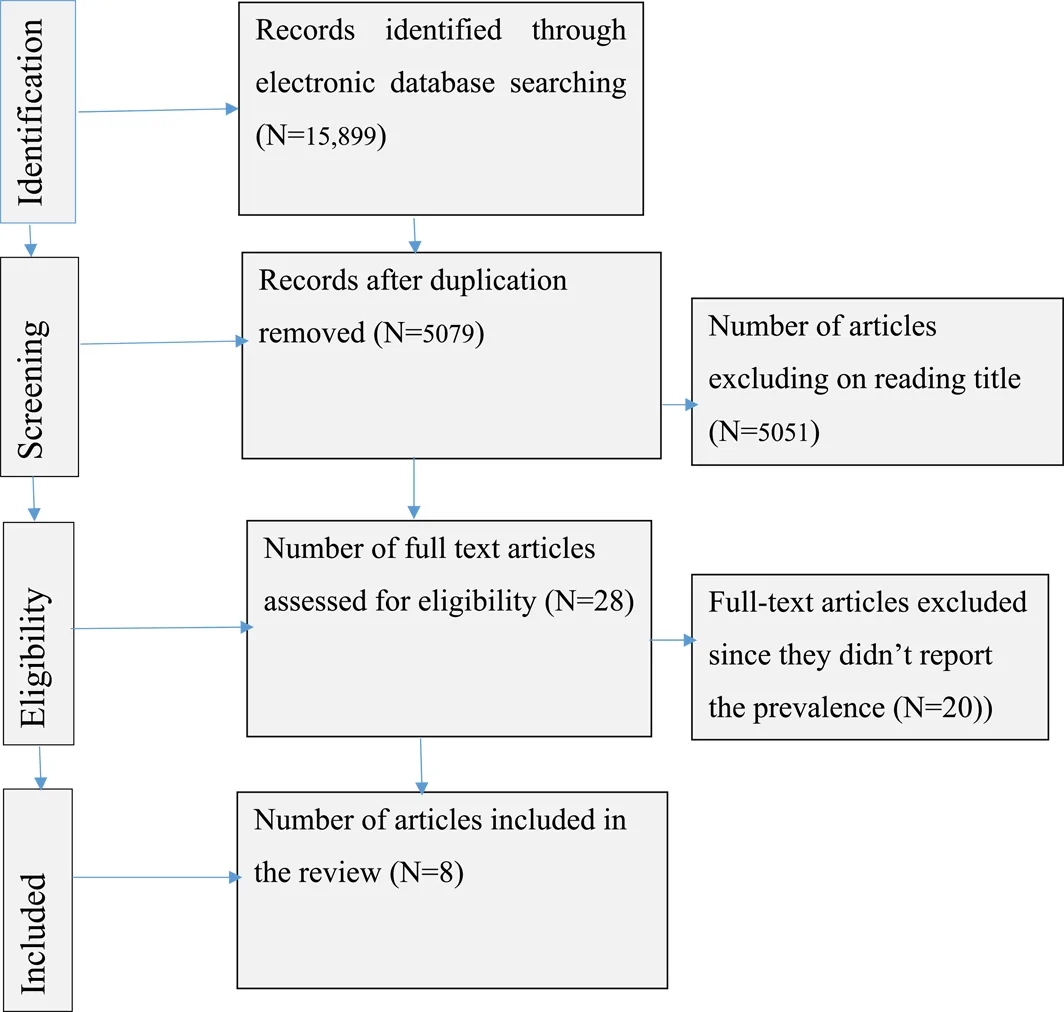
PRISMA flow diagram for the selection of studies included in systematic review and meta‐analysis of post‐stroke depression and determinants in Ethiopia (*n* = 8).

### 9.2 Study Characteristics

In this systematic review and meta‐analysis, 8 studies were included. There were between 82 and 424 participants in each study. Regarding the study design, all studies utilized a facility‐based cross‐sectional study design. Considering the region in which the included studies were conducted, four studies were from Addis Ababa (Wubshet et al. 2022; Tsehayneh and Tafesse 2020; Zewde et al. 2023; Mohammed et al. 2023), and four were from the Amhara region (Zemed et al. 2021; Eriku et al. 2023; Fantu et al. 2022; Zemed, Nigussie Chala, et al. 2021). Two studies employed systematic random sampling, and six studies used consecutive sampling as their method of sampling (Table 2).

**TABLE 2:** Detailed overview of the included studies on the prevalence of post‐stroke depression and determining factors in Ethiopia (*n* = 8).

Author	Pub year	Region	Study design	Study population	SS	Prevalence (%)	Sampling technique
Wubshet et al.	2022	Addis ababa	IBCS	Post‐stroke patients	249	27.5	Systematic random
A. Zemed et al.	2021	Amhara	IBCS	Post‐stroke patients	204	49.6	Consecutive sampling
Tsehayneh and Tafesse	2020	Addis ababa	IBCS	Post‐stroke patients	84	32.2	Consecutive sampling
Eriku et al.	2023	Amhara	IBCS	Post‐stroke patients	424	65.6	Systematic random
Fantu et al.	2022	Amhara	Case‒control	Post‐stroke patients	240	68.75	Consecutive sampling
Zewdie Y et al.	2023	Addis ababa	IBCS	Post‐stroke patients	82	12	Consecutive sampling
Mohamed et al.	2023	Addis ababa	IBCS	Post‐stroke patients	160	43.3	Consecutive sampling
Zemed et al.	2021	Amhara	IBCS	Post‐stroke patients	180	23.88	Consecutive sampling

### 9.3 Meta‐Analysis

The pooled prevalence of post‐stroke depression was 40.9% (95% CI: 26.52–55.28, *p* < 0.001). The analysis revealed significant heterogeneity among the included studies (I^2^ = 91.1%, *p* < 0.001). Consequently, the pooled prevalence of post‐stroke depression was determined using a random‐effects model, specifically the DerSimonian and Laird (D + L) method (Figure 2).

**FIGURE 2:**
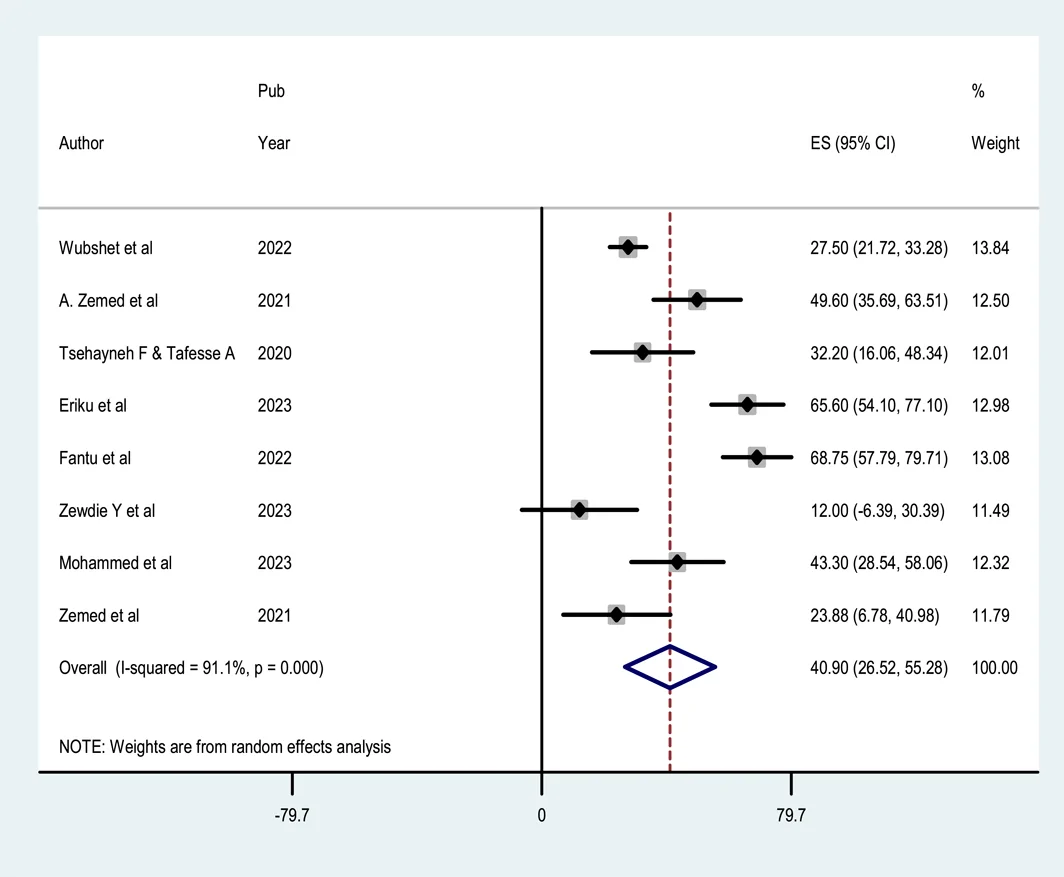
Forest plot for the pooled prevalence of post‐stroke depression in Ethiopia (*n* = 8).

### 9.4 Publication Bias

Publication bias was detected by the visual inspection of the funnel plot and statistical Egger's test. A *p*‐value less than 0.05 from Egger's test indicated notable publication bias, implying that studies with larger effects (either positive or negative) are more likely to be published. The funnel plot displayed an uneven distribution of studies, suggesting presence of publication bias. This observation was supported by Egger's test, which yielded a statistically significant *p*‐value of 0.000 at the 5% significance level, providing further evidence of publication bias.

## 10 Quality Assessment

Each of the included articles had a score of 7–8 on the quality assessment, indicating good quality. The Joanna Briggs Institute (JBI) critical appraisal checklist, which has nine items, was used to assess the quality of each of the included studies.

### 10.1 Subgroup Analysis

Given the high level of heterogeneity in this review, subgroup analysis was conducted using the study setting. The final analysis revealed that the highest prevalence of post stroke depression was reported in the Amhara region (52.89% CI: 35.18, 70.60), and a low prevalence was reported in Addis Ababa (29.29% CI: 19.20, 39.38) (Figure 3).

**FIGURE 3:**
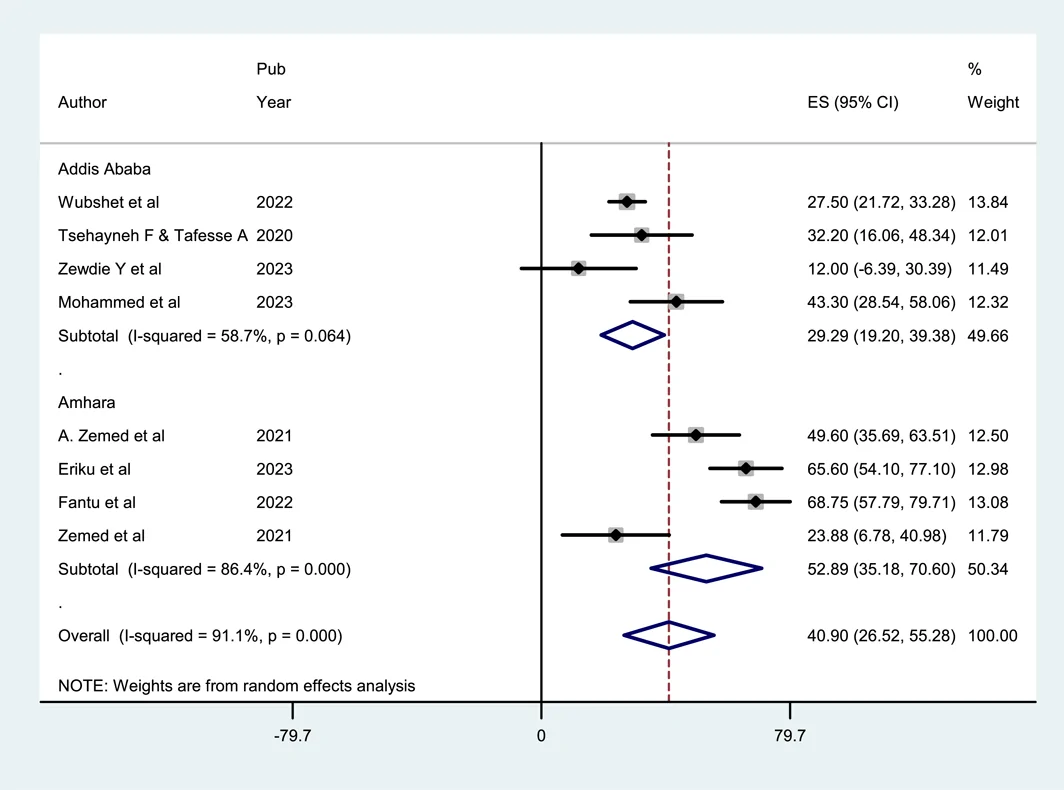
Forest plot showing subgroup analysis using the study setting.

The subgroup analysis revealed that studies utilizing systematic random sampling showed the highest pooled prevalence of post stroke depression, which is 46.21% (95% CI: 8.88–83.54, I^2^ = 97%, *p*‐value = 0.000) (Figure 4).

**FIGURE 4:**
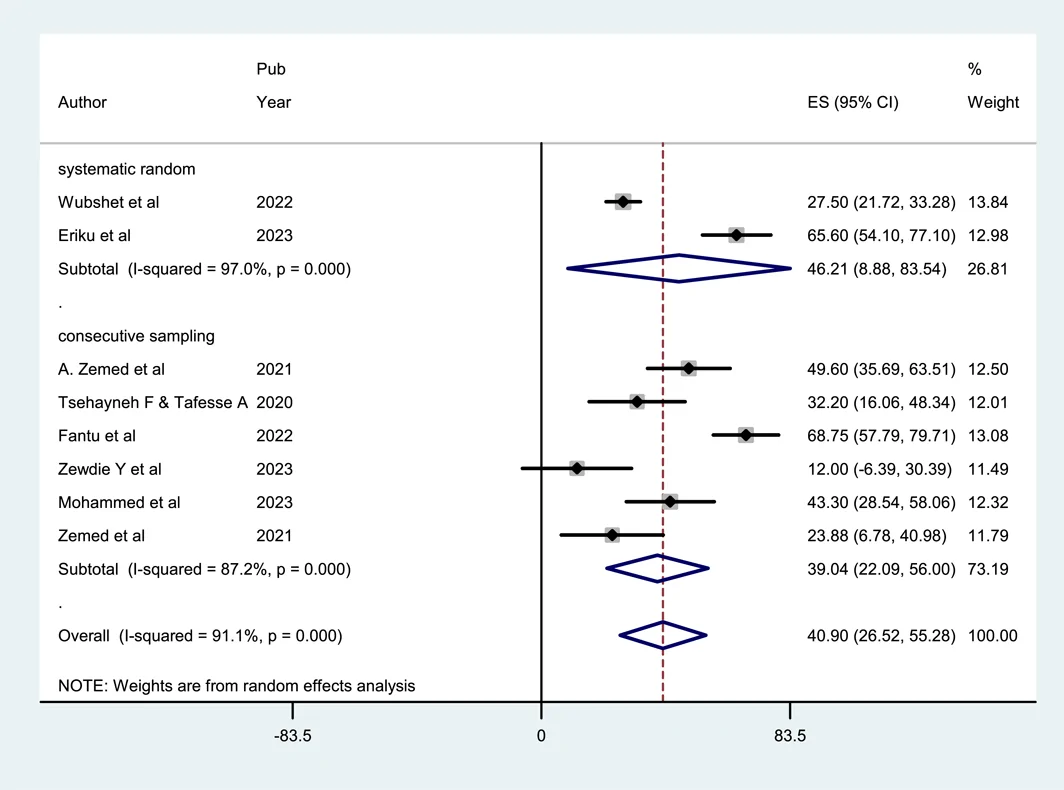
Forest plot showing subgroup analysis using systematic random sampling.

## 11 Determinants of Post‐Stroke Depression

The findings showed that female stroke patients were 3.51 times more likely (AOR 3.51; 95% CI, 1.87–6.58) to experience post‐stroke depression compared to male patients (Figure 5).

**FIGURE 5:**
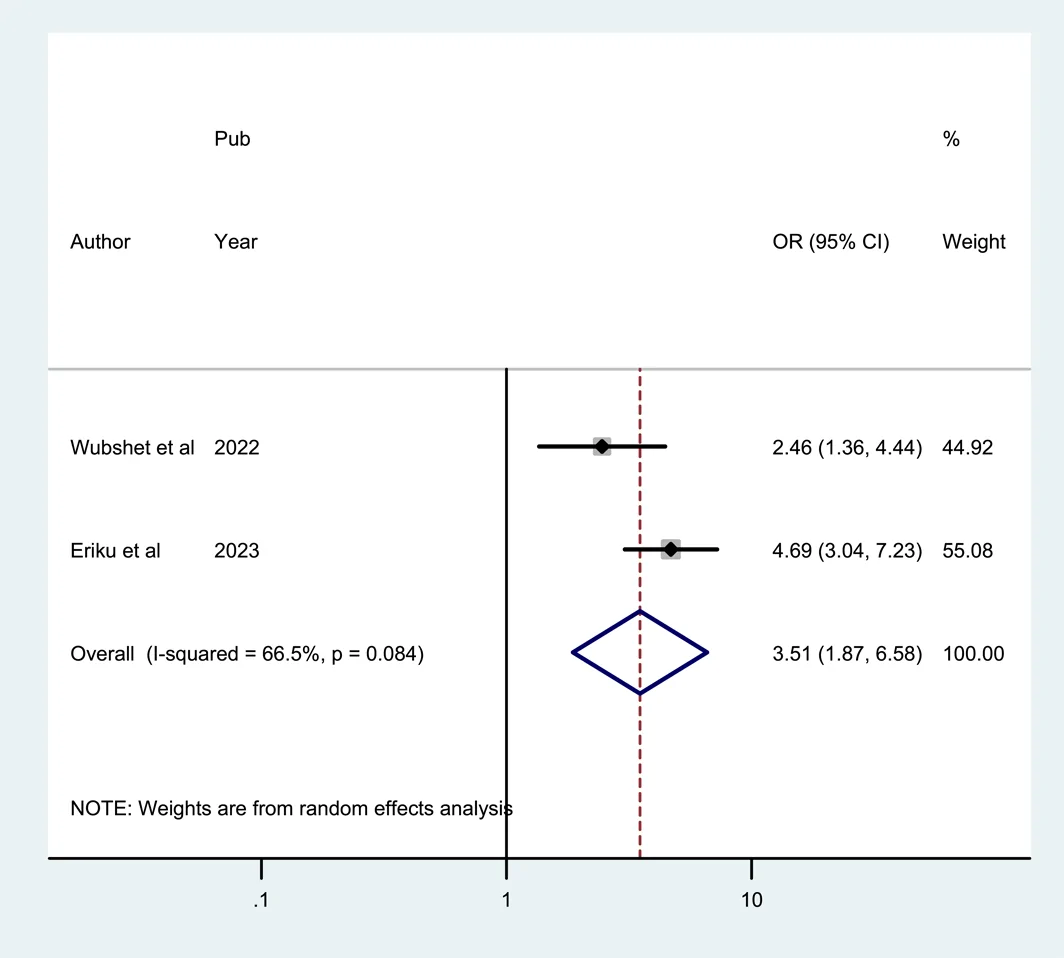
Forest plot showing the association between gender and post‐stroke depression.

Stroke patients with no formal education had a 2.02 times higher likelihood (AOR 2.02; 95% CI, 1.29–3.17) of experiencing post‐stroke depression compared to those with a college degree or higher (Figure 6).

**FIGURE 6:**
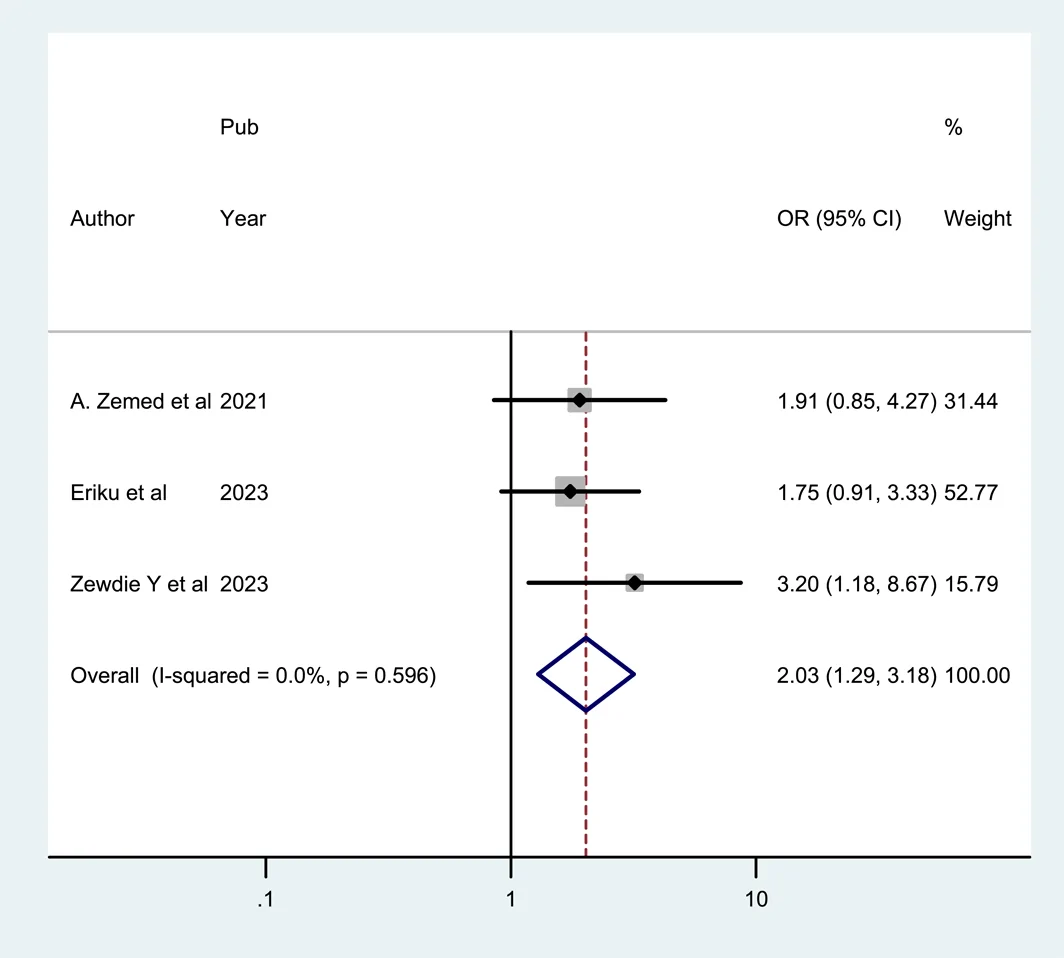
Forest plot showing the association between educational level and post‐stroke depression.

The odds of developing depression among patients over the age of 40 were 1.59 times more likely compared with patients younger than 40 (AOR = 1.59, 95% CI, 1.09, 2.32) (Figure 7).

**FIGURE 7:**
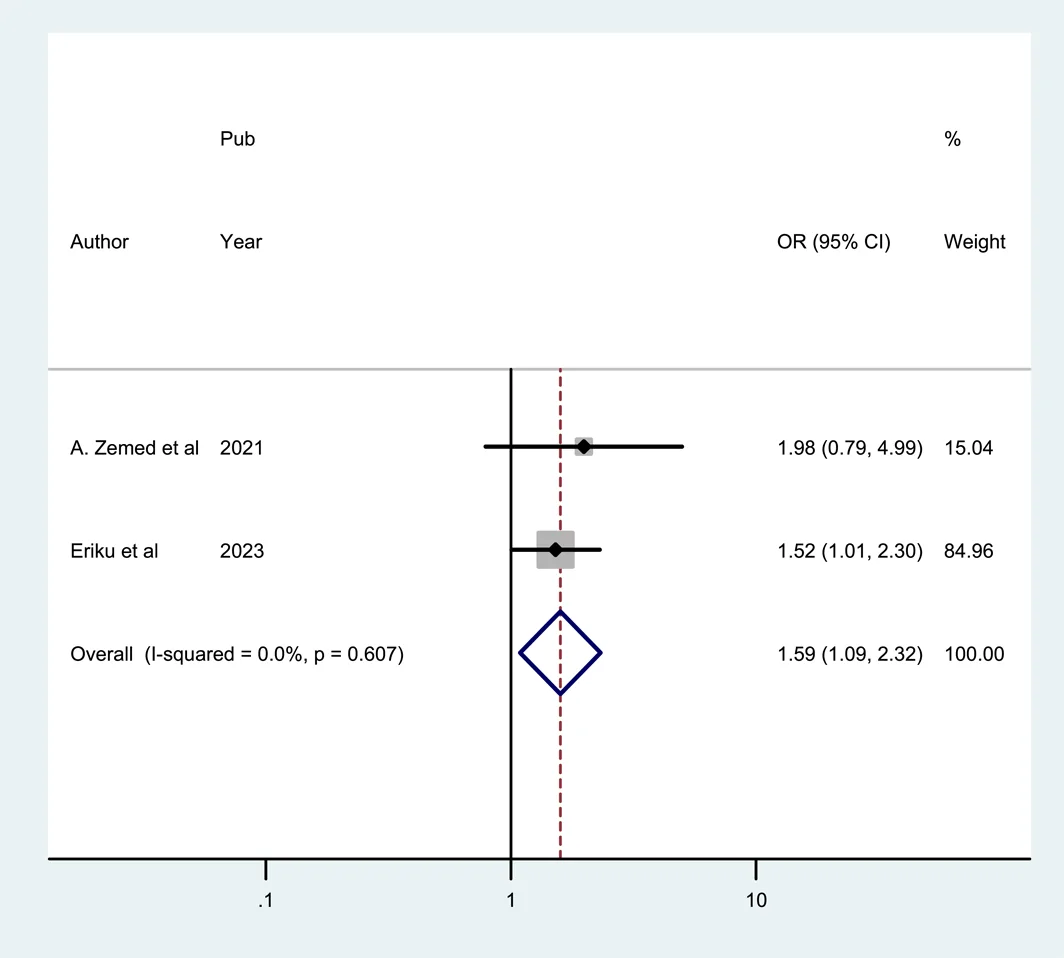
Forest plot showing the association between patient age and post‐stroke depression.

## 12 Discussion

In Ethiopia, the pooled prevalence of post stroke depression was 40.9% (95% CI: 26.52–55.28). The result of the current study is in line with previous findings of studies done in Iran (45% (Ebrahimi‐Rad et al. 2016), 47.4% (Hasanzadeh et al. 2014), and 49% (Dalvand et al. 2018) and Finland 48.1% (Berg et al. 2003). However, the current pooled prevalence of PSD is higher than result of studies conducted in the USA (31%) (Barker‐Collo 2007), Germany (31.1%) (Schöttke and Giabbiconi 2015), Ghana (36.5%) (Sarfo et al. 2017) and Nigeria (25.5%) (Oladiji et al. 2009). This difference may be attributed to variation in the study population, such as differences in depression screening tool, socio‐demographic factors, or the severity of depression considered. The discrepancy of results might be due to the assessment tool, study design, sample size, the health care delivery system, economic status, and life style. Whereas, the finding of this study was lower than the study conducted in Bangladesh 70% (Wada et al. 2005). This discrepancy may stem from the use of different assessment tools in the above study. The Bangladesh study employed the Hamilton Depression Rating Scale (HDRS), while the primary studies in our review used the Patient Health Questionnaire (PHQ‐9). Other tools, such as the Beck Depression Inventory (BDI) and the Hospital Anxiety and Depression Scale (HADS), were also used in some other studies to screen stroke patients for depression. Additionally, variations in the age range of participants and the timing of stroke onset could offer further plausible explanations.

Female stroke patients were more likely to experience PSD compared to male patients. This finding is similar with the previous studies (Ojagbemi et al. 2017; Loh et al. 2017). Although there is not enough evidence to conclude why women are more likely than men to experience depression. The possible justification might be due to women's numerous duties and obligations, such as child care, meal preparation, and homework, will raise their vulnerability to depression, especially in Ethiopian women. And also, the hormonal changes after premenstrual and menopausal transition, which are associated with a decrease in estrogen, may be another potential factor that would increase the risk of developing depression (Albert 2015; Bartels et al. 2013).

In addition, stroke patients with no formal education had higher likelihood of experiencing post‐stroke depression compared to those with a college degree or higher. This finding is supported by a studies conducted in Italy and the USA (Paolucci et al. 2005; Whyte and Mulsant 2002). The reasons could be that, although stroke is a major contributor to severe disability and a low quality of life, individuals without formal education may not be aware of the poor prognosis, potential complications, or psychosocial and economic effects of the condition. As a result, they may be unable to conduct timely depression screenings, potentially raising the risk of depression. The odds of developing depression among patients over the age of 40 were more likely compared with patients younger than 40. This was consistent with the previous study conducted in Malaysia (Omar et al. 2021). Since older individuals often spend much of their lives alone and experience social isolation, it can be argued that social connections are essential for survival and well‐being. The high rates of depression are closely associated with feelings of social isolation and loneliness (Zhang et al. 2023).

### 12.1 Strength and Limitations

As a strong point, the authors used some of the important major databases to search for original articles. This systematic review and meta‐analysis is not free from limitations. Although, articles were searched from all region of Ethiopia, only a subset of regions is represented which limits the generalizability of the findings to the entire country. Moreover, most studies included were cross‐sectional, limiting the ability to assess causality or observe longitudinal trends. Substantial statistically significant heterogeneity was observed across studies which undermines the pooled estimate of post stroke depression suggests that chance could be responsible for between‐study variability. Finally, the study excluded gray literature which leads for publication bias.

One limitation of this study is the small number of studies available for assessing factors associated with post‐stroke depression. The included studies varied in terms of the factors investigated, study populations, and definitions and measurements of the potential associated factors, which limited the comparability of the findings. Therefore, the results regarding factors associated with post‐stroke depression should be interpreted with caution and should not be considered definitive. Further studies with larger sample sizes, standardized definitions, and comparable measures of potential associated factors are needed to provide more robust evidence.

## 13 Conclusion and Recommendations

According to the current review, post stroke depression is highly prevalent in Ethiopia. Being female, being illiterate and being over 40 years old were factors associated with post stroke depression. The results of this study have several clinical implications for health services and policymakers. Although important gaps in terms of the most effective treatment for PSD remain, a greater awareness on depression after stroke and its impact on survival among patients, families, and health professionals, could facilitate recognition, earlier treatment, and improved.

Outcomes. In addition, attention should be given to strengthening social support; health professionals should focus on reducing disability/physical dependency and depression, as these are vital factors for improving quality of life. There is urgent need for integration of screening for and management of post stroke depression. Hence, screening all post stroke patients with different screening tools is practical, and further studies are needed to assess the validity of these screening tools and also to assess PSD as a predictor of stroke outcome. Physicians and physical therapists caring for stroke patients should pay particular attention to the mental health of women, patients older than 40 years, and illiterate.

**Abbreviations**
AORs: Adjusted Odds Ratio
IBCS: Institutional‐Based Cross‐Sectional Study
PSD: Post‐stroke Depression

## Author Contributions

**Getanew Kegne Nigatie:** conceptualization, investigation, writing – original draft, methodology. **Berhanu Wale Yirdaw:** formal analysis, software, visualization. **Getaw Wubie Assefa:** conceptualization, methodology, software. **Worku Chekol Tassew:** investigation, validation, formal analysis.

## Funding

The authors have nothing to report.

## Ethics Statement

Ethical approval was not required for this systematic review and meta‐analysis because the study used data exclusively from previously published studies and did not involve the recruitment of human participants, primary data collection, or access to identifiable personal information. Therefore, institutional ethical approval was not required. Ethics approval ID: Not applicable. Ethics approval institution: Not applicable. The review was conducted in accordance with relevant ethical principles and the Preferred Reporting Items for Systematic Reviews and Meta‐Analyses (PRISMA) guidelines.

## Consent

The authors have nothing to report.

## Conflicts of Interest

The authors declare no conflicts of interest.

## Data Availability

All relevant data are within the manuscript and its Supporting Information files.
